# State of the Patients in the Bristol Dispensary, from the 1st of May to the 1st of June, 1801

**Published:** 1801-07

**Authors:** 


					4 State of Diseases in the Bristol Dispensary.
State of the Patients in the Brijlol Difpenfary, from the \Ji of May
to the iji of June, I 801.
Typhus Fever - - - 34
Dvfenteria and Diarrhoea - 4
Podagra ------ 1
Co; lumption - - - -. 9
Cough and Dyfpnoea - - 2
Afthma ------ 2
Peripneumonia - - - 6
Afcarides - - - 1
Afthenia ----- 4
Rheumatifm - - - - 6
Dropfy - - 5
Menftrual Complaints - - 4
Febris Simplex - - - - 8
Gnftrodynia and Cardialgia 4
Paralyfis ----- 3
Nervous : - - - - 2
Bilious ----- 1
I&erus - - - - I
Eryfipelas - - - -/ - I
Variola - - - - - - 1

				

